# Sensing Capacity in Dysprosium Metal–Organic Frameworks Based on 5-Aminoisophthalic Acid Ligand

**DOI:** 10.3390/s22093392

**Published:** 2022-04-28

**Authors:** Javier Cepeda, Isabel Blasco-Pascual, Sara Rojas, Duane Choquesillo-Lazarte, Francisco J. Guerrero-Arroyo, Diego P. Morales, Jose Ángel García, Antonio Rodríguez-Diéguez, Alfonso Salinas-Castillo

**Affiliations:** 1 Department of Applied Chemistry, Faculty of Chemistry, University of the Basque Country (UPV/EHU), 20018 Donostia-San Sebastián, Spain; javier.cepeda@ehu.eus; 2 Department of Analytical Chemistry, Faculty of Science, University of Granada, 18071 Granada, Spain; isablasco@ugr.es; 3 Department of Inorganic Chemistry, Faculty of Science, University of Granada, 18071 Granada, Spain; srojas@ugr.es (S.R.); javiguearr98@gmail.com (F.J.G.-A.); 4 Laboratorio de Estudios Cristalográficos, IACT, CSIC-UGR, Av. Las Palmeras nº4, 18100 Granada, Spain; duane.choquesillo@csic.es; 5 Pervasive Electronics Advanced Research Laboratory (PEARL), Department Electronics and Computer Technology, University of Granada, 18071 Granada, Spain; diegopm@ugr.es; 6 Departamento de Física Aplicada II, Facultad de Ciencia y Tecnología, Universidad del País Vasco/Euskal Herriko Unibertsitatea (UPV/EHU), 48940 Leioa, Spain; joseangel.garcia@ehu.eus

**Keywords:** MOF, dysprosium, 5-aminoisophthalic, sensing, luminescence

## Abstract

Two novel metal-organic frameworks (MOFs), based on dysprosium as the metal and the 5-aminoisophthalic acid (5aip) ligand, have been solvothermally synthesized, with the aim of studying and modulating their luminescence properties according to the variation of solvent in the structure. These materials display intense photo-luminescence properties in the solid state at room temperature. Interestingly, one fascinating sensory capacity of compound **2** regards obtaining a variation of the signal, depending on the solvent to which it is exposed. These results pave the way for a new generation of sensitive chemical sensors.

## 1. Introduction

Dysprosium ions have been widely employed as fascinating magnetic and luminescent centers for constructing coordination compounds, due to their large magnetic moment and significant single-ion anisotropy, derived from spin−orbit coupling and the crystal field effect and the characteristic narrow fluorescence emission, in such a way that it is the optimal ion to construct interesting materials for exhibiting its sensing capabilities [1]. This is particularly true for metal–organic frameworks (MOFs), which are crystalline coordination polymers with a large structural versatility that have a wide range of applications [2]. These materials are synthesized by mixing metal ions (acting as the nodes of the framework) with organic molecules (which play the role of links) to form potentially porous architectures that are often extended over two or three dimensions. Among them, dysprosium-based MOFs (Dy-MOFs) have been less widely studied, owing to the large size of the Dy(III) ion, because the usual high coordination numbers it presents impose distorted symmetries that introduce too much coordination flexibility into the crystal-building, a fact that somehow prevents the prediction of the resulting topology of the framework. The study of lanthanide-based MOFs has been traditionally linked to fields such as luminescence [3], gas adsorption [4], optical storage [5], and magnetism [6]. In particular, our research group and others are focusing on this point in order to further analyze the luminescent properties of these materials [7]. In this regard, in recent years, several Dy-MOFs based on dicarboxylic ligands, showing interesting magnetic and luminescent properties, have been designed and synthesized by our group [8]. With the aim of continuing this task, we decided to explore and prepare dysprosium-based MOFs by introducing small structural modifications into the ligands to compare their luminescent properties. Therefore, in the present work, we are making use of 5-aminoisophthalic (5aip) acid as the ligand due to its great variety of coordination modes, a characteristic that makes it very useful for obtaining a diversity of systems [9].

The main challenge in sensor research can be summarized by the following expectations: (a) the search and selection of the appropriate materials, as improvement and novelty in the recognition mechanisms necessary for the instantaneous identification of analytes, and the improvement of the mechanism to create the signal to be obtained from the sensor; (b) the development of new materials as matrices that effectively immobilize the receptor molecules and, in this way, achieve stability and reproducibility in the sensors; (c) the design of systems to develop automation in the use of sensors and their implementation in new technologies. The conventional analysis procedures used in many cases are expensive, slow, complex, and dependent on high-cost infrastructures and trained personnel for their use.

Paper-based analytical devices (PADs) offer powerful platforms for the design of chemical analysis platforms due to their advantages, such as a lower cost per test, the reduction of the assay time and volume consumption, eco-friendly support, and, in general, simple strategies in terms of food and environmental monitoring [10,11]. On account of these merits, PADs are capable of integrating with a sensing system based on an optical signal (luminescence or color) [12], Along with these attractive features and promising prospects, the development and implementation of nanomaterials in these systems are necessary for their future application [13].

Bearing in mind the latter point, we report on the synthesis and structural characterization of two new MOFs that exhibit luminescent properties; specifically, one of them shows interesting sensing capacities, depending on the solvent to which it is exposed.

## 2. Materials and Methods

### 2.1. Chemicals

All chemicals were of reagent grade and were used as commercially obtained without any further purification. N,N-dimethylformamide anhydrous (HCON(CH_3_)_2_, ≥99.8%), dysprosium(III) chloride hexahydrate (DyCl_6_·6H_2_O, ≥99.99%), 5-aminoisophthalic acid (H_2_NC_6_H_3_-1,3-(CO_2_H)_2_, 94%), methanol (CH_3_OH, 99.8%), ethanol (C_2_H_5_OH, 99.8%), dimethyl sulfoxide anhydrous (C_2_H_6_OS, ≥99.9%), and acetic acid (CH_3_COOH, 99–100%) were purchased from Sigma Aldrich (Darmstadt, Germany). All the aqueous solutions were prepared with ultrapure water (0.22 μS/cm, 25 °C, MilliQ©, Millipore, Burlington, CA, USA).

Cellulose filter paper (Ref. 1240, sheet 203 × 254 mm, base weight 85 g/m^2^; thickness 200 µm; retention 14–18 µm) was obtained from Filter-Lab (Barcelona, Spain).

### 2.2. Synthesis of Compound ***1***

A DMF:H_2_O mixture (1 mL, 1:1) containing DyCl_3_·6H_2_O (0.035 mmol, 13.9 mg) was slowly added to another DMF:H_2_O solution (1 mL, 1:1) of the 5aip ligand (0.055 mmol, 10 mg). The resulting mixture was poured into a glass vessel of 8 mL capacity and sonicated for 2 min to obtain a homogeneous solution, then it was closed with a screw cap and placed in an oven at 95 °C. Soft pink crystals, formed from compound **1**, were grown after 1 day. They were filtered, washed with water, and dried. Yield: 15%, based on the metal.

### 2.3. Synthesis of Compound ***2***

The procedure is similar to the previous one but it involved doubling the concentration of the initial reagents. The vessel was closed with a screw cap and placed in an oven at 95 °C. Soft pink crystals corresponding to compound **2** were grown after 1 day of solvothermal reaction of the reagent mixture in an oven at 95 °C. The crystals were also filtered, washed with water, and dried. Yield: 21%, based on the metal.

### 2.4. X-ray Diffraction Data Collection and Structure Determination

Crystals for the compounds were mounted on glass fiber and were used for the data collection on a Bruker D8 Venture, with a photon detector equipped with graphite monochromated MoKα radiation (λ = 0.71073 Å). The data reduction was performed with the APEX2 [14] software and was corrected for absorption using SADABS [15]. The crystal structures were established by direct methods using the SIR97 program [16] and refined by full-matrix least-squares on F^2^, including all reflections, using anisotropic displacement parameters by means of the WINGX crystallographic package [17,18]. Generally, anisotropic temperature factors were assigned to all atoms except for hydrogen atoms, which were riding their parent atoms with an isotropic temperature factor arbitrarily chosen to be 1.2 times that of the respective parent. The final R(F), wR(F2), goodness-of-fit agreement factors, details on the data collection, and analysis can be found in Table 1. Crystallographic data for the structures reported in this paper have been deposited with the Cambridge Crystallographic Data Centre under the supplementary publication nos. CCDC 2103353 and 2103354 for compounds. Copies of the data can be obtained free of charge on application to the Director, CCDC, 12 Union Road, Cambridge, CB2 1EZ, UK (fax: +44-1223-335033; e-mail: deposit@ccdc.cam.ac.uk).

### 2.5. Photoluminescence Measurements

Photoluminescence spectra were recorded on an Edinburgh Instruments FLS920 (Edinburgh, UK) spectrometer equipped with a closed-cycle helium cryostat. All measurements were performed under a high vacuum (of ca. 10−9 mbar) to avoid the presence of oxygen or water in the sample holder. For steady-state measurements, an IK3552R-G HeCd (GoPhotonics, Zurich, Switzerland) continuous laser (325 nm) was used as an excitation source, whereas a Müller-Elektronik-Optik SVX1450 (Aldridge, UK) Xe lamp was employed to collect the excitation spectra. The luminescence collected in the UV-vis region was analyzed with a photomultiplier tube (PMT) coupled to the spectrometer. However, the decay curves were measured using a µF900 microsecond pulsed lamp (Edinburgh, UK) or a pulsed laser diode LDH-P-C-375 (PicoQuant, Berlin, Germany) (λ = 375 nm), depending on the emission longevity.

### 2.6. Preparation of the Test Paper

The PAD was fabricated using a craft-cutting technique as a cost-efficient, simple and reproducible process; the pattern of the µPAD was first designed using Illustrator software (Adobe Systems, San Jose, CA, USA) and the design was cut with a CO_2_ laser engraver (Rayjet, Barcelona, Spain). To be specific, disks (8 mm in diameter) of cellulose-grade filters were first produced using a cutting technique that was optimized using a sheet of paper. The device was prepared by drop-casting the necessary reagents in each sensing area; then, 15 µL of MOFs was dispensed onto the device, then dried at room temperature for 5 min, and stored in the dark until use under ambient atmospheric conditions. Then, 15 μL solvent solution in water at different concentrations was dropped onto the manufactured test paper and then, we proceeded to measure the fluorescence spectra.

The fluorescence spectra of PAD were obtained using a Cary Eclipse UV-Vis fluorescence spectrophotometer in the range of 400–550 nm, with a xenon lamp as the excitation source. Luminescence intensity was measured using the following conditions: λ_ex_ = 325 nm; the photomultiplier voltage was set at 700 V; excitation and emission slits of 5 nm were used.

## 3. Results and Discussion

The hydrothermal reactions of the 5-aminoisophthalic acid with dysprosium chloride at different concentrations (10 mL) at 95 °C for 48 h produced prismatic crystals of different MOFs. The crystal structures of the compounds were determined using single-crystal X-ray diffraction.

### 3.1. Description of the Structures

**Figure 1:**
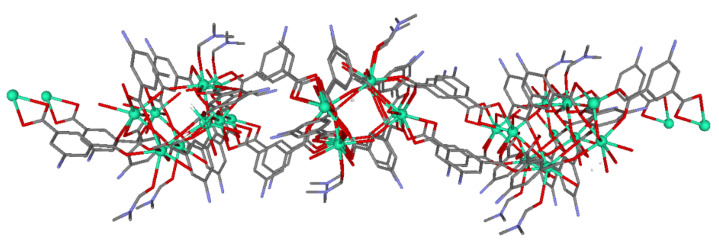
The two-dimensional structure of compound **1**. Crystallization molecules and hydrogen atoms are omitted for clarity. Color code: C = gray, Dy = green, N = blue, O = red.

Compound **1** crystallized in the triclinic space group *P*-1. It consists of a 2 D-layered structure composed of Dy(III) dimeric units, established by the coordination of carboxylate groups pertaining to different 5aip ligands (Figure 1). The asymmetric unit of this material is composed of four dysprosium atoms, six dianionic ligands, three coordination water molecules, two coordination DMF molecules, six lattice water molecules, and one lattice DMF molecule. The dysprosium ions are bridged by different linkers, to initially generate dimeric entities that are further linked to one another by means of both of the other carboxylate groups pertaining to the 5aip ligands, forming the dimer and the rest of the 5aip ligands coordinating through the edges of the dimer. The resulting two-dimensional network possesses cavities that are occupied by DMF and water molecules within it. The four dysprosium atoms are crystallographically independent, where all show DyO_8_ coordination polyhedra except for the Dy2 atom, which exhibits a DyO_7_ polyhedron.

The Dy1 atom presents a coordination polyhedron, established by eight oxygen atoms belonging to six different 5aip linkers. Instead, the DyO_7_ coordination environment of the Dy2 atom is built by five oxygen atoms of five different dianionic 5aips, one water molecule (O12), and one DMF molecule. The main difference between Dy3 and Dy4 atoms with respect to Dy1 is the presence of solvent molecules in their coordination environment. That is to say, the Dy3 atom contains one oxygen atom (O50) of a coordination water molecule, while the Dy4 atom possesses one water (O20) and one DMF terminal ligand. The Dy–O_carb_ bond distances are in the range of 2.234(7)–2.596(6) Å, among which the Dy–O_water_ distances are around 2.3 Å. Within the dimers, it must be highlighted that the intradinuclear Dy⋯Dy distance, which involves Dy1 and Dy4 atoms, is 3.9120(7) Å. Finally, the layers are packed close to one another by means of hydrogen bonds, established between the amino groups of 5aip ligands and solvent molecules, among which the former act as hydrogen-bonding donors and the latter as acceptors. In spite of the presence of voids among the layers, the 2D-layered structure did not remain stable enough to allow the removal of solvent molecules during its thermal activation, which led to an amorphous product that could not be further studied (see Supplementary Information).

**Figure 2:**
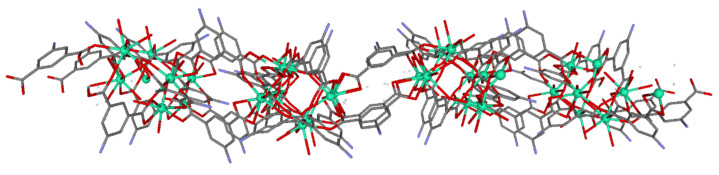
Bidimensional structure for compound **2**. Crystallization molecules and hydrogen atoms are omitted for clarity. Color coding: grey = C, green = Dy, blue = nitrogen, red = oxygen.

Compound **2** crystallized in the triclinic space group of *P*-1. The 2D-MOF structure is composed similarly of Dy(III) dimers connected through the carboxylate groups of different 5-amino-1,3-benzenedicarboxylic ligands, generating a bidimensional coordination polymer (Figure 2). The asymmetric unit of this material is composed of four dysprosium atoms, six dianionic ligands, five coordination water molecules, nine crystallization water molecules, and two crystallization DMF molecules. These dysprosium atoms are bridged by different linkers, generating a two-dimensional network with big cavities that are occupied by crystallization DMF molecules and water molecules. The four dysprosium atoms are crystallographically independent, both having DyO_8_ coordination polyhedra except in the case of dysprosium 1, which has a DyO_7_ polyhedron.

The coordination environment of the Dy1 atom is built by five oxygen atoms, pertaining to five different 5aip linkers, and completed by two oxygen atoms (O37 and O40) of water molecules. The Dy2 atom has a DyO_8_ chromophore, with eight oxygen atoms of six different dianionic linkers and two solvent molecules. On the other hand, the Dy3 and Dy4 atoms show similar DyO_8_ environments that are distinguished from Dy2 by the fact that they possess one (O31) and two (O32 and O41) coordination water molecules, respectively. The Dy–O_carb_ bond distances are of the same order as in compound **1**, where the intradinuclear Dy⋯Dy distance is 3.9089(3) Å for the Dy2⋯Dy4 bridge. Finally, the layers are packed close to one another by means of hydrogen bonding interactions among the amino groups of ligands, crystallization DMF molecules, and water molecules. The principal difference between the two compounds is that in compound **2**, the DMF molecules coordinate with dysprosium atoms, whereas in compound **1,** there are no coordinated DMF molecules. The potentially porous structure of compound **2** presents a continuous release of the molecules occupying the pores, mixed with the coordination solvent molecules (see Supplementary Materials), a fact that precludes its adsorption capacity in a similar way to that mentioned for compound **1.**

### 3.2. Luminescence Properties

**Figure 3:**
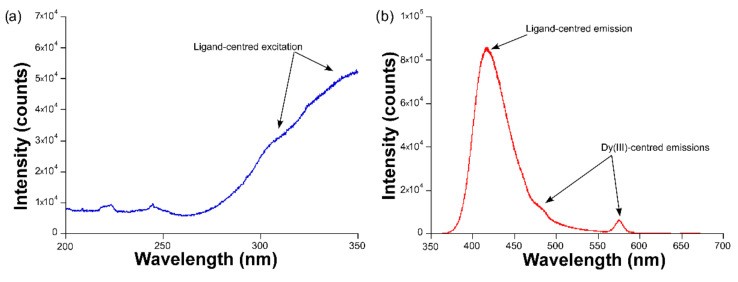
The (**a**) excitation (λ_em_ = 576 nm) and (**b**) emission (λ_ex_ = 325 nm) spectra of compound **1**, acquired at room temperature.

The luminescence properties of compounds **1** and **2** were first studied in the solid state by measuring the excitation and emission spectra at room temperature over the polycrystalline samples. The emission spectrum of compound **1,** measured under monochromated laser irradiation (λ_ex_ = 325 nm), is dominated by a wide band covering the 400–500 nm range (Figure 3), which may be attributed to the ligand fluorescence, in view of the similar profile shown by the free ligand, as described in a previous study [19]. In particular, the band may be assigned to the π ← π* transitions occurring at the aromatic ring of the ligand, as previously shown for other compounds based on the 5aip ligand [20,21]. In any case, this band also presents a minor shoulder at around 483 nm, in addition to another band of similar intensity sited at 576 nm. According to reports regarding other previous Dy-based compounds [22], these two signals correspond to the main intraionic transitions of the ion, namely, the ^6^H_15/2_ ← ^4^F_9/2_ and ^6^H_13/2_ ← ^4^F_9/2_, respectively, for λ_em_ = 483 and 576 nm. The excitation spectrum, measured by monitoring the latter, shows a wide band where no narrow maxima attributed to the intraionic excitations of Dy(III) are observed, meaning that the Dy(III)-centered emission is preferentially occurring through ligand absorption, i.e., the antenna effect.

**Figure 4:**
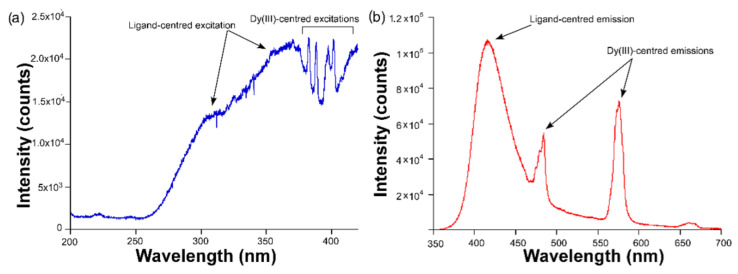
The (**a**) excitation (λ_em_ = 576 nm) and (**b**) emission (λ_ex_ = 325 nm) spectra of compound **2,** acquired at room temperature.

Although consisting of the same ligand, when the ligand is coordinated to Dy(III) ions to form the structure of compound **2**, the Dy(III)-centered emission is significantly improved with regard to **1**, which finding may be corroborated by the relative increase in the intensity of the bands corresponding to the intraionic transitions peaking at λ_em_ = 483 and 576 nm (Figure 4). Moreover, the stronger luminescence of this compound also allows the observation of a third multiplet sited at λ_em_ = 665 nm, which can be attributed to the ^6^H_11/2_ ← ^4^F_9/2_ transition. Among the three Dy(III)-centered bands, the second one is the most intense, as usually happens for most of the coordination polymers analyzed in previous studies [23,24,25].

**Figure 5:**
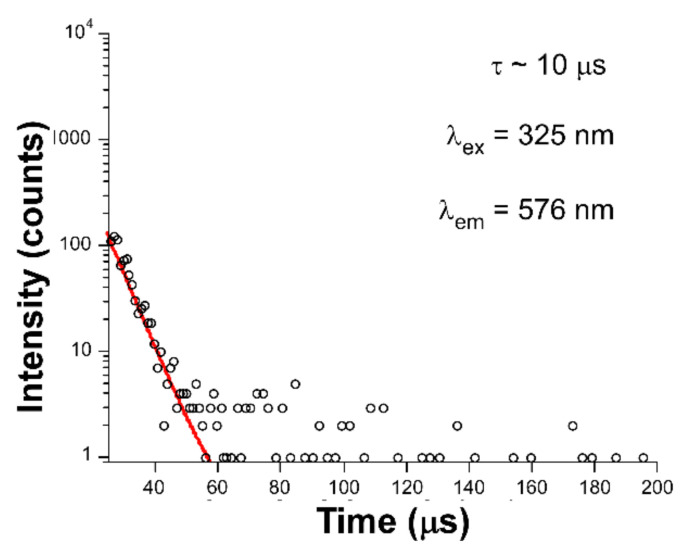
Decay curve with the best fitting for compound **2**.

In good agreement with the previous statements, the excitation spectrum of **2** also presents numerous narrow bands corresponding to the intraionic bands of Dy(III) ion, through which excitation takes place from the ground ^4^F_9/2_ term to the excited states. Paying attention to the crystal structure of both compounds, the lower intensity shown by compound **1** may be attributed to the presence of the DMF molecules coordinated to the metal center. As is well known, the occurrence of certain molecules possessing C–H, O–H, and N–H bonds, which behave as oscillators that are coupled to the energy gaps existing between the excited and ground terms of the lanthanide, may act as effective quenchers of the lanthanide-centered luminescence [26]. In this way, part of the charge populating the excited levels (^4^F_9/2_, in this case) may be transferred to these vibrations activating the substantial non-radiative pathways responsible for the weak luminescence of compound **1**, a fact that is not so marked in compound **2**. Inspired by the better emission properties of compound **2**, the decay curves were measured under the same wavelengths (λ_ex_ = 325 and λ_em_ = 576 nm). The fitting of the curve (Figure 5) by means of a conventional exponential equation [I_t_ = A_0_ + A_1_exp(−t/τ_1_)] gave a short lifetime value of 10 µs, which may be considered a similar value to other Dy(III)-based compounds [7,27].

**Figure 6:**
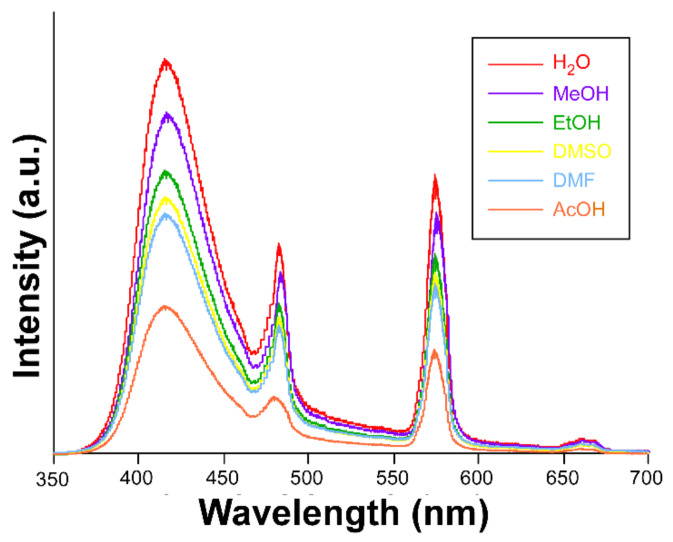
The emission spectrum of compound **2**. Inset: color coding.

The good emissive properties and potential porosity of compound **2,** given the presence of lattice solvent molecules enclosed in the cavities of the structure, encouraged us to seek deeper insights into the sensing capacity of the material. To that end, several suspensions, starting from 3 mg of the compound in various solvents ((H_2_O, MeOH, EtOH, DMF, DMSO, and acetic acid (AcOH)) were analyzed by measuring the emission spectra, keeping the aforementioned experimental setup (λ_ex_ = 325 nm). It is important to note that the compound retains its structural integrity after being dispersed in H_2_O and AcOH, as corroborated by the PXRD collected for samples filtered from these experiments (see Figure S2 in the Supplementary Materials). As can be inferred from Figure 6, the dispersion of the solid in the solvents does not promote significant changes in the emission profile (compared to the solid state), although it does cause a drop in the overall emission capacity. The signal turns off in the following order: H_2_O > MeOH > EtOH > DMSO > DMF > AcOH, which follows the same trend for the polarity of the solvents. This trend is in good agreement with the lower capacity of the solvent to create and keep the excitons active upon UV light absorption as the polarity of the solvent decreases. This fact has been previously observed for many other lanthanide-based complexes [28,29]. Taking into account the fact that this compound does not behave as a permanently porous framework, in view of its thermal behavior, it may be speculated that the quenching mechanism might involve the interactions taking place between the quenchers and the external face of the crystalline particles.

### 3.3. Test Paper

To be specific, disks (8 mm in diameter) of cellulose filter paper were first produced using a cutting technique that was optimized using a sheet of paper (203 × 254 mm) for the production of 396 replicas (see Figure S5 in the Supplementary Materials).

**Figure 7:**
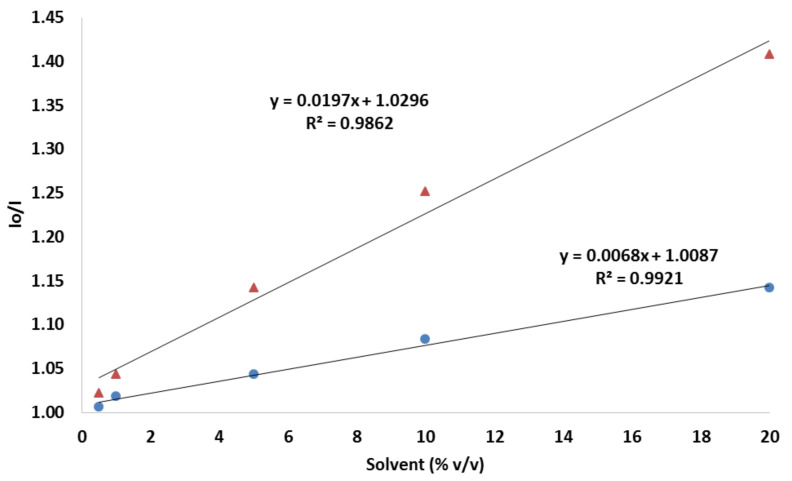
Calibration curve for acetic acid (orange triangle) and ethanol (blue dot) using a test paper with compound **2**.

As mentioned, the fluorescence intensity of PAD paper is quenched when the solvent is presented. The decrease in fluorescence can be quantified by correlating the ratio of initial intensity (Io) and the intensity (I) of the paper with the solvent present in the sample (see Figure 7 and Figure S6 in the Supplementary Materials). The experimental dependence between the Io/I value and the solvent (%) is in linear dependence. The regression equation is Io/I = 0.0197C + 1.03, with R^2^ = 0.9862 for acetic acid and Io/I = 0.0068C + 1.0087, with R^2^ = 0.9921. The detection limit of the solvent was calculated, in light of the 3σ/slope laws, to be as low as 0.30% for acetic acid and 0.88% for ethanol. In addition, the quantification limit was calculated with the 10 σ/slope equation, resulting in 1.02% and 2.94% for acetic acid and ethanol, respectively. The repeatability as a relative standard deviation, obtained using 10 different PADs at 10% (*v*/*v*) of acetic acid, was 4.2%, an acceptable precision considering the measuring system used. The lifetime of the PAD was studied for 20 days in two cycles of the prepared device, regularly checking their responses to a 10% (*v*/*v*) acetic acid. During the study period, it was observed that there was no decreasing signal; the response of the device was stable.

Considering that the concentration-dependent evolution of both quenchers, in the form of Stern-Volmer plots, fitted well to a linear equation, other processes, such as intersystem crossing, the formation of charge transfer complexes, and static/dynamic quenching can be discarded, while the major contribution ruling the quenching mechanism seems to be the polarity of the solvents.

## 4. Conclusions

Two novel metal–organic-frameworks (MOFs), based on dysprosium as the metal and the 5-aminoisophthalic acid ligand, have been solvothermally synthesized, with the aim of studying and modulating their luminescence properties according to the variation of the solvent in the structure. These materials display intense photo-luminescence properties in their solid state at room temperature. Both compounds exhibit the main characteristic emission of the ligand, whereas the Dy(III)-based luminescence at room temperature is comparatively weak, given that the energy transfer from the ligand to the lanthanide ion is not efficient. However, compound **2** presents better properties, although both compounds share the same ligand, a fact that may be related to the absence of coordinated DMF molecules. The dispersion of the solid in the solvents causes a drop in the total emission capacity, following the same trend for the polarity of the solvents. In the same way, this system works in paper-based devices, opening the doors to future uses and the implementation of these compounds in portable, cheap, and environmentally sustainable systems.

## Figures and Tables

**Table 1:** Crystallographic data and structure refinement details for compounds.

	Compound 1	Compound 2
CCDC	2103354	2103353
Formula	C_57_H_75_Dy_4_N_9_O_39_	C_54_H_70_Dy_4_N_8_O_39_
M	2160.26	2105.18
Crystal System	Triclinic	Triclinic
Space group	*P*-1	*P*-1
*T* (K)	100	100
*a* (Å)	12.0914 (16)	13.5278 (7)
*b* (Å)	13.4186 (17)	14.2508 (8)
*c* (Å)	24.493 (3)	19.8040 (10)
*α* (deg)	76.929 (4)	77.679 (2)
*β* (deg)	85.627 (4)	70.471 (2)
*γ* (deg)	87.332 (5)	83.739 (2)
*V* (Å^3^)	3858.0 (8)	3512.4 (3)
Z	2	2
Density (g cm^−3^)	1.86	1.991
*μ* (mm^−1^)	3.927	4.31
Observed reflections	10617	56588
*R_int_*	0.092	0.0435
R_1_ ^b^/wR_2_ ^c^ (I > 2σ(I))	0.0553/0.1279	0.0260/0.0513
R_1_ ^b^/wR_2_ ^c^ (all data)	0.0783/0.1409	0.0366/0.0544
GoF (S) ^a^	1.029	1.048
Largest diff. pk and hole (eÅ^−3^)	3.355 and −3.289	0.872 and −0.555

^a^ S = [Σw(F_0_^2^ − F_c_^2^)^2^/(N_obs_ − N_para_m)]^1/2^. ^b^ R_1_ = Σ||F_0_|–|F_c_||/Σ|F_0_|. ^c^ wR_2_ = [Σw(F_0_^2^ − F_c_^2^)^2^/ΣwF_0_^2^]^1/2^ w = 1/[σ^2^(F_0_^2^) + (aP)^2^ + bP] where P = (max(F_0_^2^,0) + 2F_c_^2^)/3.

## Supplementary Materials

The following supporting information can be downloaded at: https://www.mdpi.com/article/10.3390/s22093392/s1, Figure S1: Comparison of the PXRD data of compound **1**; Figure S2: Comparison of the PXRD data of compound **2**; Figure S3: Thermogravimetric profiles of compounds **1** and **2**; Figure S4: View along a (left), b (middle) and c (right) axis of compound **1** (above) and compound **2** (below); Figure S5: Paper analytical device development; Figure S6: Spectrum emission using the test paper, with compound **2** for acetic acid (A) and ethanol (B). Conditions for fluorescence excitation at 325 nm, with intensity based (Io/I) at a maximum peak emission of 410 nm.
