# Supplementary material for: Sensing Capacity in Dysprosium Metal–Organic Frameworks Based on 5-Aminoisophthalic Acid Ligand
Source: Sensors (Basel). 2022 Apr 28;22(9):3392. doi: 10.3390/s22093392 (PMC9103290; doi:10.3390/s22093392)
Supplement: Supplementary file 1 [file sensors-22-03392-s001.zip › sensors-1569947-supplementary.pdf]

Supporting Information for:

Sensing Capacity in Dysprosium Metal–Organic Frameworks Based on 5-Aminoisophthalic Acid Ligand

Javier Cepeda [1], Isabel Blasco-Pascual [2], Sara Rojas [3], Duane Choquesillo-Lazarte [4], Francisco J. Guerrero-Arroyo [3], Diego P. Morales [5], Jose Ángel García [6], Antonio Rodríguez-Diéguez [3,*] and Alfonso Salinas-Castillo [2,*]

1 Department of Applied Chemistry, Faculty of Chemistry, University of the Basque Country (UPV/EHU), 20018 Donostia-San Sebastián, Spain.

2 Department of Analytical Chemistry, Faculty of Science, University of Granada, 18071 Granada, Spain.

3 Department of Inorganic Chemistry, Faculty of Science, University of Granada, 18071 Granada, Spain.

4 Laboratorio de Estudios Cristalográficos, IACT, CSIC-UGR, Av. Las Palmeras nº4, 18100 Granada, Spain.

5 Pervasive Electronics Advanced Research Laboratory (PEARL), Department Electronics and Computer Technology, University of Granada, 18071, Granada, Spain.

6 Departamento de Física Aplicada II, Facultad de Ciencia y Tecnología, Universidad del País Vasco/Euskal Herriko Unibertsitatea (UPV/EHU), 48940 Leioa, Spain.

Index:

S1. PXRD data for compounds 1 and 2.

S2. Thermogravimetric measurements.

S1. PXRD data for compounds 1 and 2.

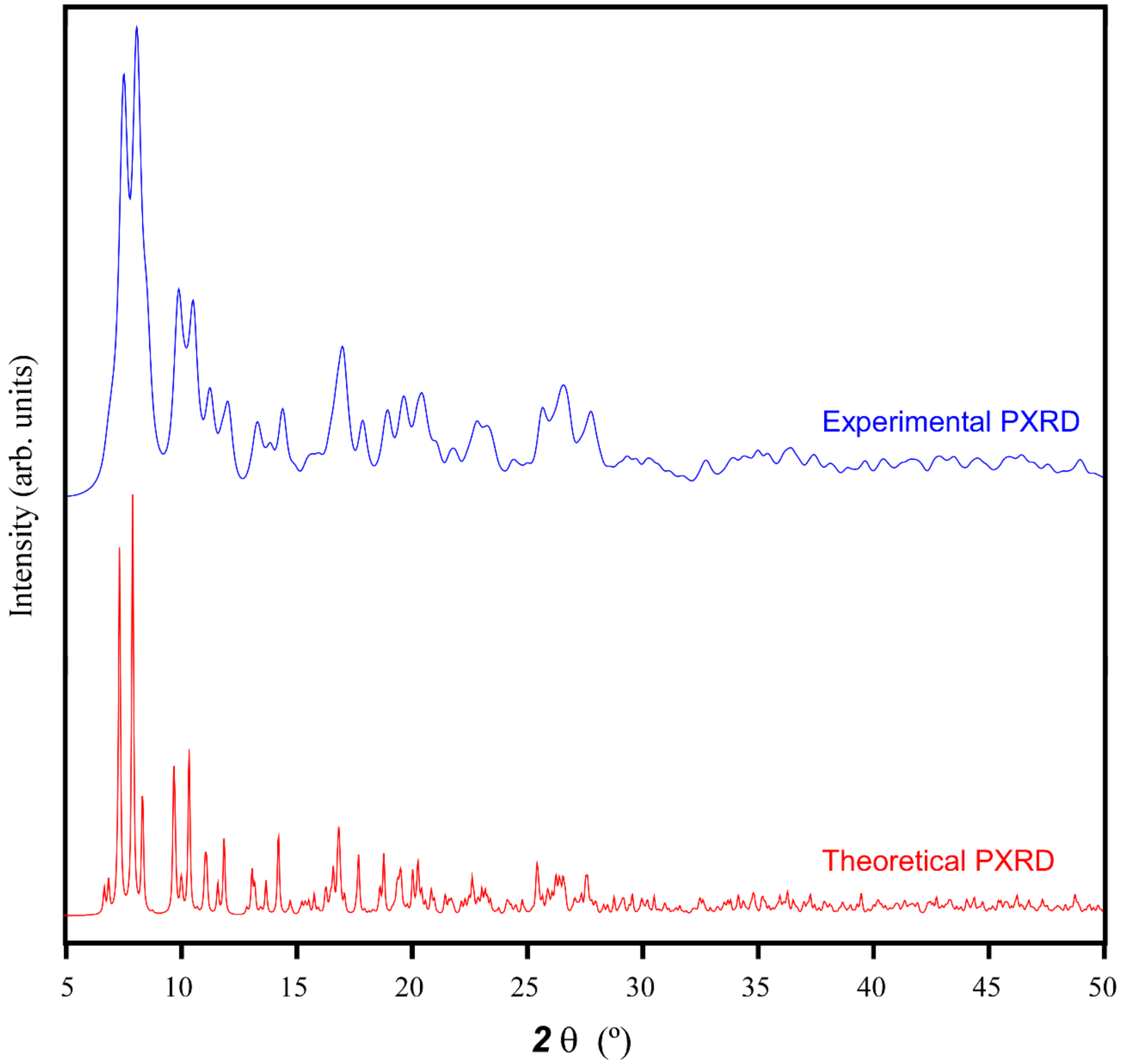

Figure S1. Comparison of the PXRD data of compound **1**.

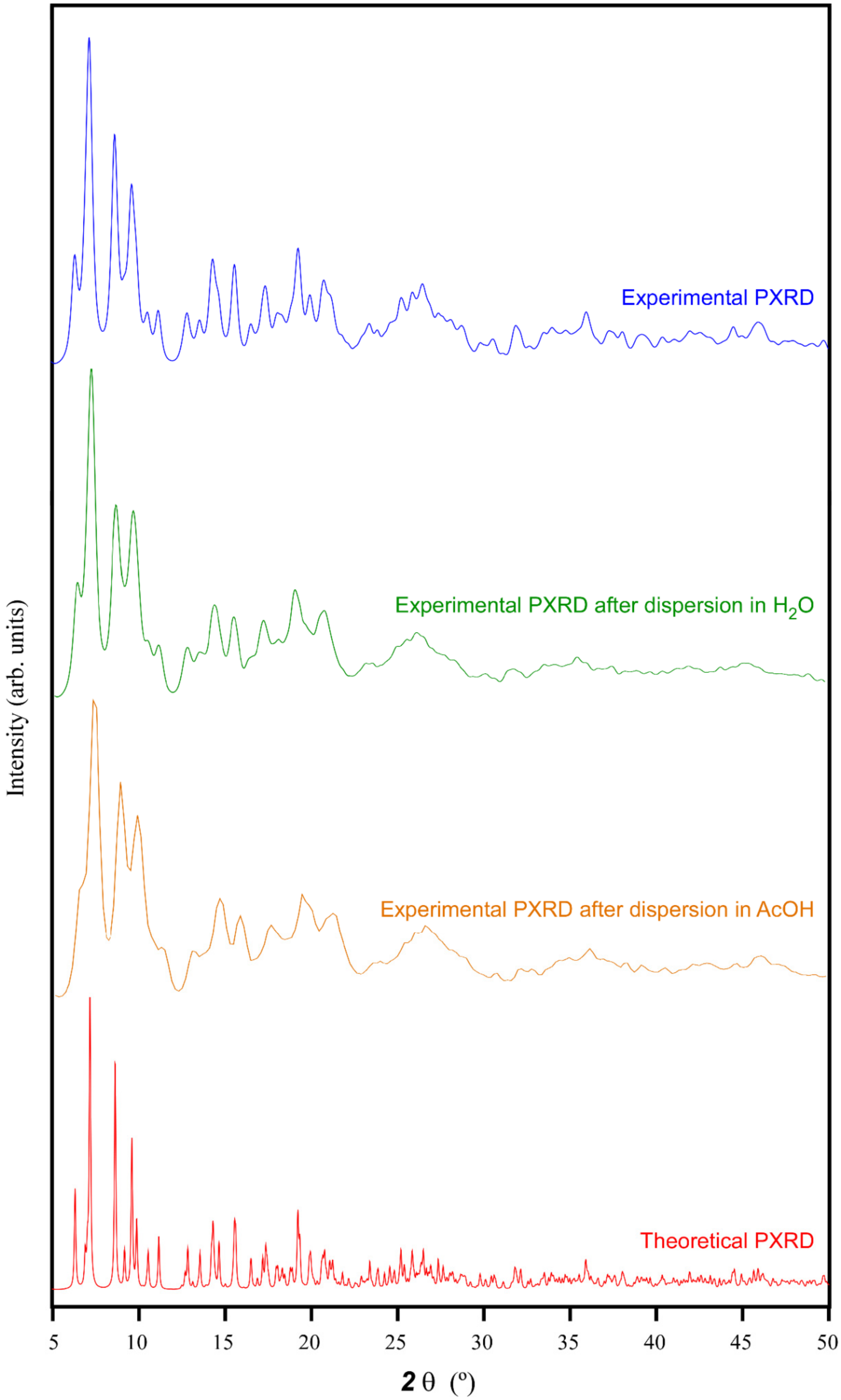

Figure S2. Comparison of the PXRD data of compound **2**.

S2. Thermogravimetric measurements.

The thermogravimetric data of compounds **1** and **2** reveal a distinct decomposition behavior that is in agreement with their crystal structure. On the one hand, compound **1** presents a first mass loss, corresponding to the lattice solvent molecules, between 40 and 250 ºC. Thereafter, a small plateau appears up to 420 ºC, above which the 2D-layered structure starts to decompose, giving rise to Dy_2O_3 as the final residue. Despite the fact that the 2D-layered structure retains its integrity (referred to as the mass percentage) between 250 and 420 ºC of temperature range, the partially desolvated compound is completely amorphous, meaning that the ordered structure of the MOF is lost upon heating, a fact that precludes its further activity as a gas adsorbent. On the other hand, compound **2** shows two steps corresponding to the loss of the lattice water molecules (in the 30–120 ºC range) and to the release of the lattice DMF and coordination water molecules (in the 130–420 ºC range). In this case, the temperature at which the structure could be activated (when voids would be empty) is not clear given the absence of a plateau. All efforts to correctly activate the MOF by heating it gave rise to amorphous solids; therefore, no further analysis of its porosity was performed.

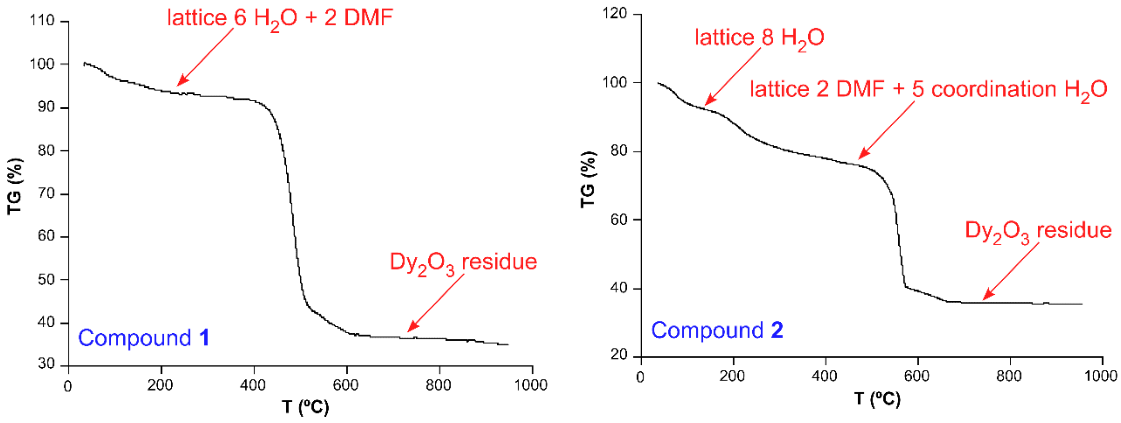

Figure S3. Thermogravimetric profiles of compounds **1** and **2**.

View along the a, b and c axes.

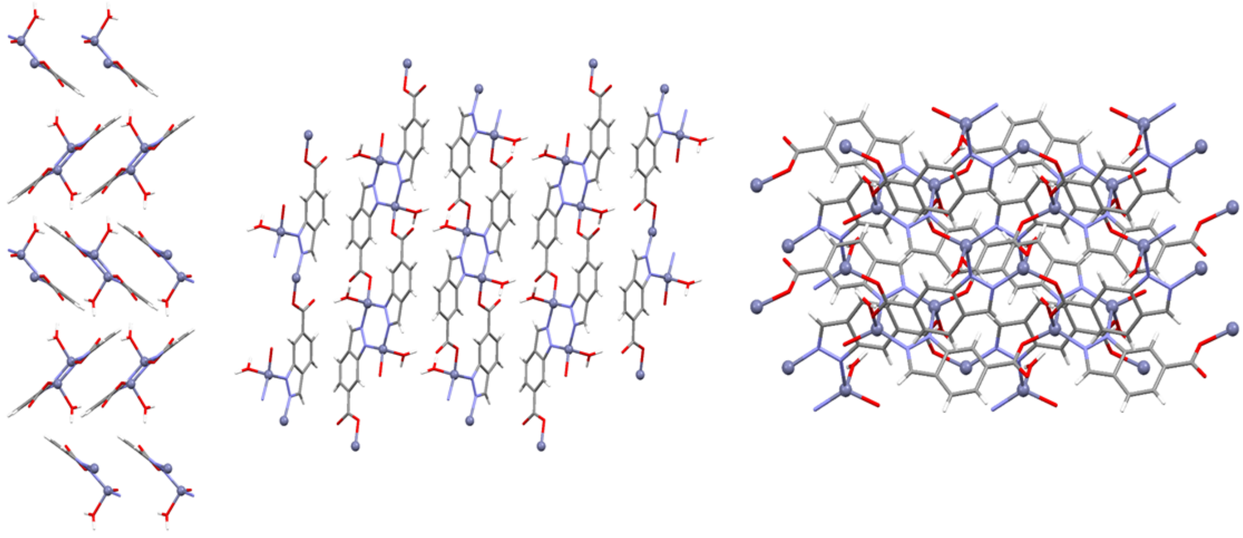

Compound 1

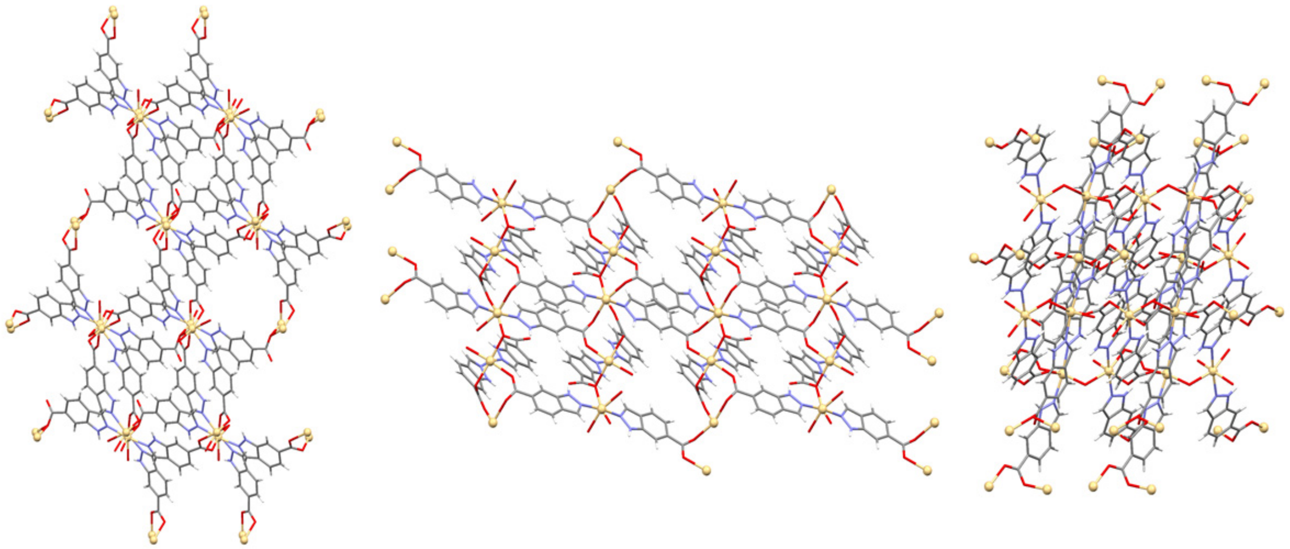

Compound 2

Figure S4. View along a (left), b (middle) and c (right) axis of compound **1** (above) and compound **2** (below).

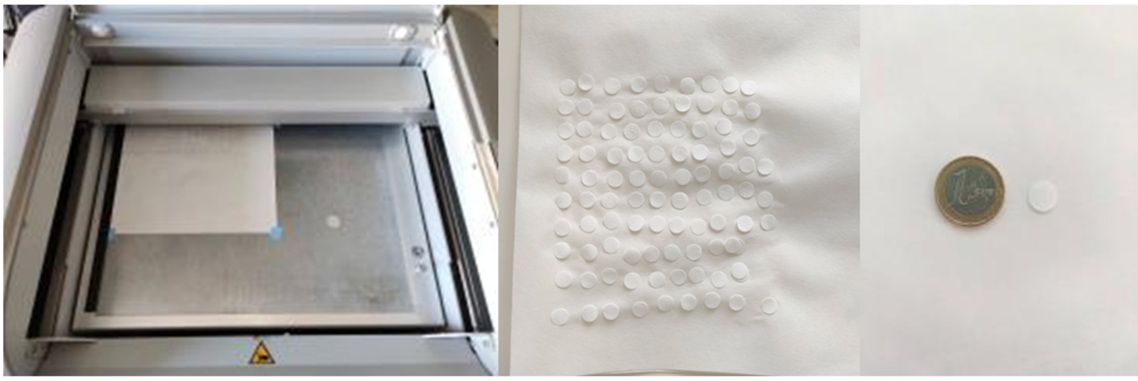

Figure S5. Paper analytical device development

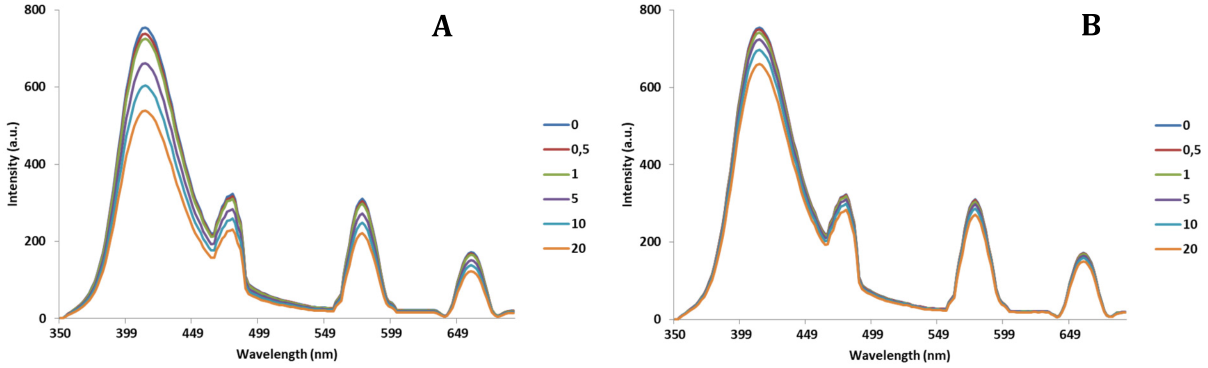

C

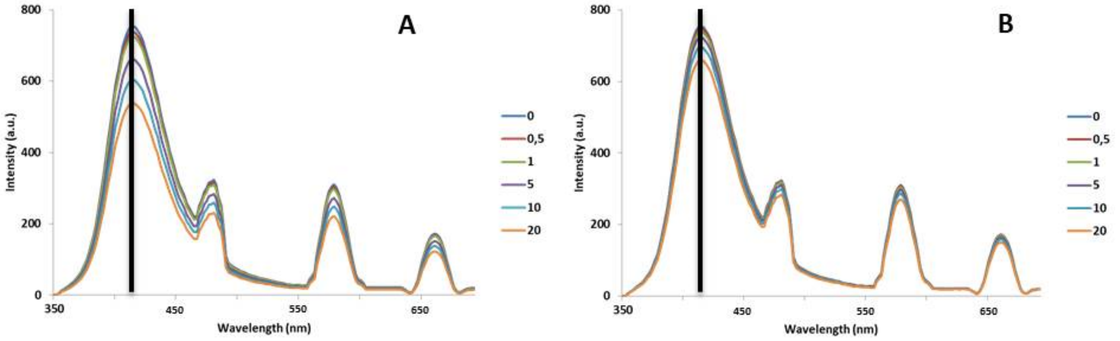

	Ethanol	Acetic acid	Ethanol	Acetic acid
0	754	754	Io/I	Io/I
0,5	749	738	1,01	1,02
1	741	725	1,02	1,04
5	723	661	1,04	1,14
10	696	603	1,08	1,25
20	660	538	1,14	1,40

Figure S6. Spectrum emission using the test paper, with compound **2** for acetic acid (**A**) and ethanol (**B**). Conditions for fluorescence excitation at 325 nm, with intensity based (Io/I) at a maximum peak emission of 410 nm (**C**).
